# Supplementary material for: Climate change is associated with higher phytoplankton biomass and longer blooms in the West Antarctic Peninsula
Source: Nat Commun. 2024 Aug 2;15:6536. doi: 10.1038/s41467-024-50381-2 (PMC11297178; doi:10.1038/s41467-024-50381-2)
Supplement: Supplementary file 1 — Supplementary Information [file 41467_2024_50381_MOESM1_ESM.pdf]

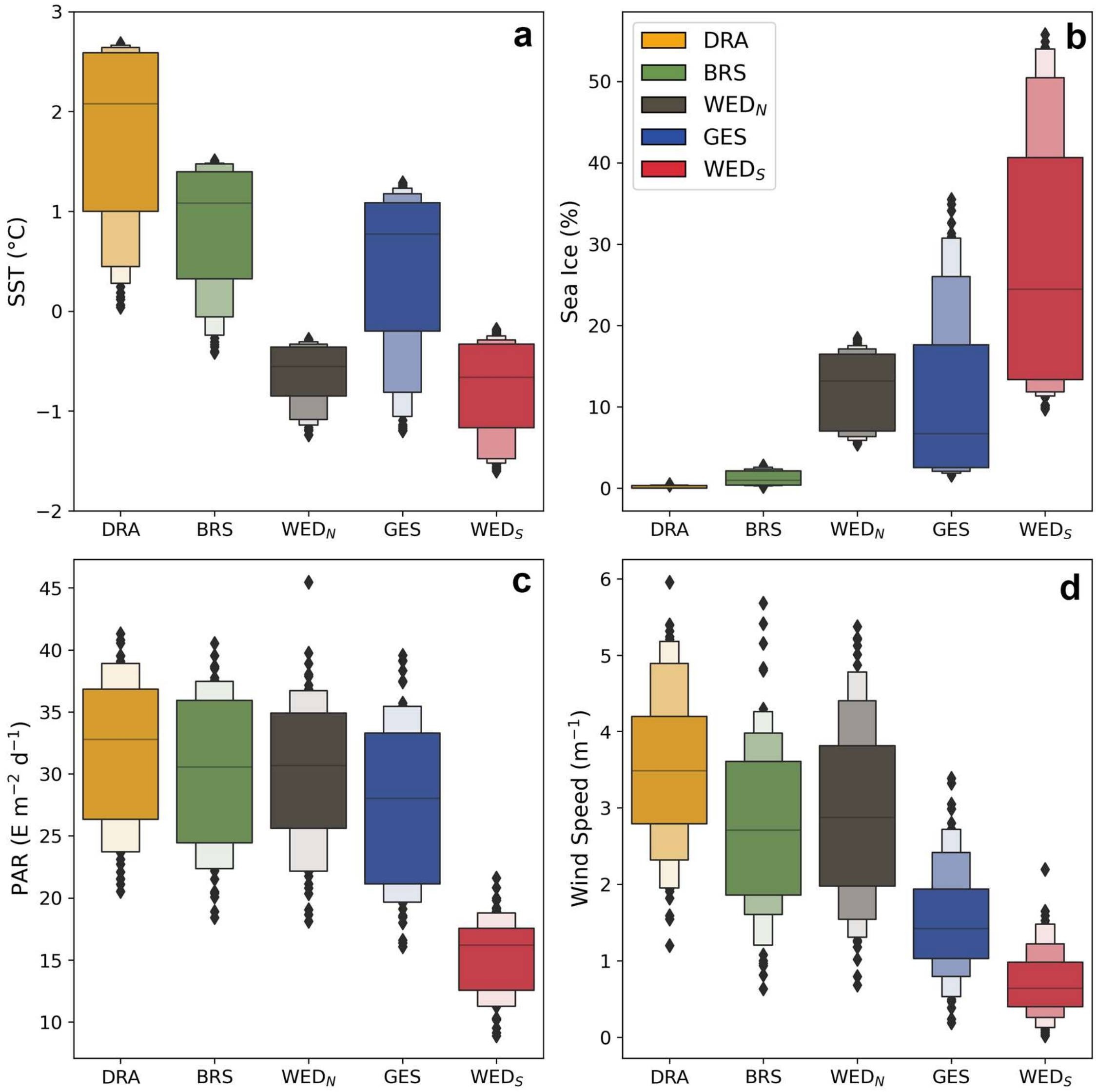

Sup. Fig. 1 | Summary of summer environmental conditions across the Antarctic Peninsula. Letter-value plots for of sea surface temperature (SST; ºC) (a), sea ice concentration (%) (b), photosynthetically active radiation (PAR; E m^{-2} d^{-1}) (c), and wind speed (ms^{-1}) (d) values in the summer (December-February) during 1998-2022 for each region. The center line of the box plots correspond to the median.

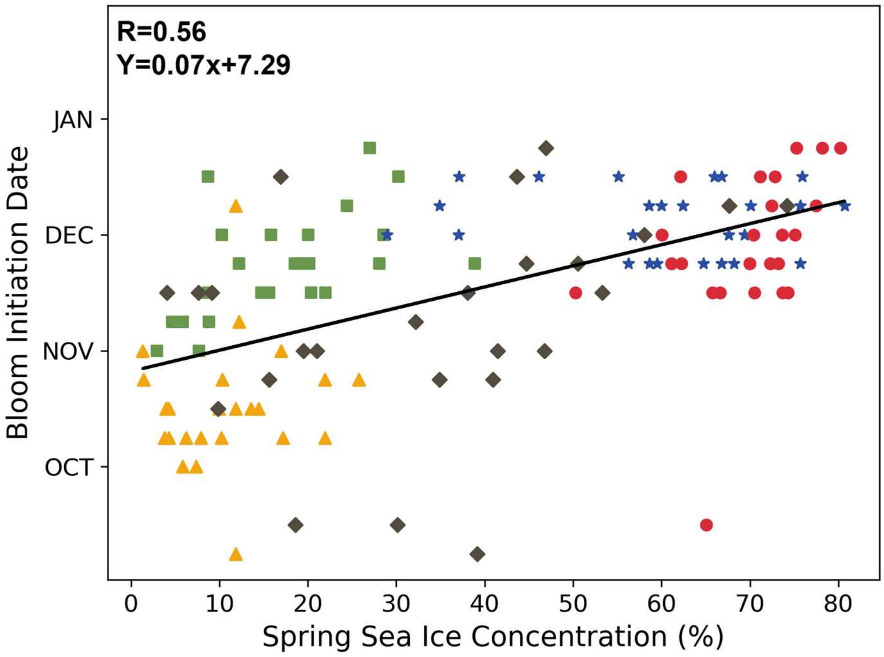

Sup. Fig. 2 | Linear relationship between bloom initiation timing and spring sea ice concentration. Linear regression between the phytoplankton bloom initiation date and the mean spring (September-November) sea ice concentration (%) during 1998-2022 for each region. The relationship is statistically significant (N=125; p-val<0.05).

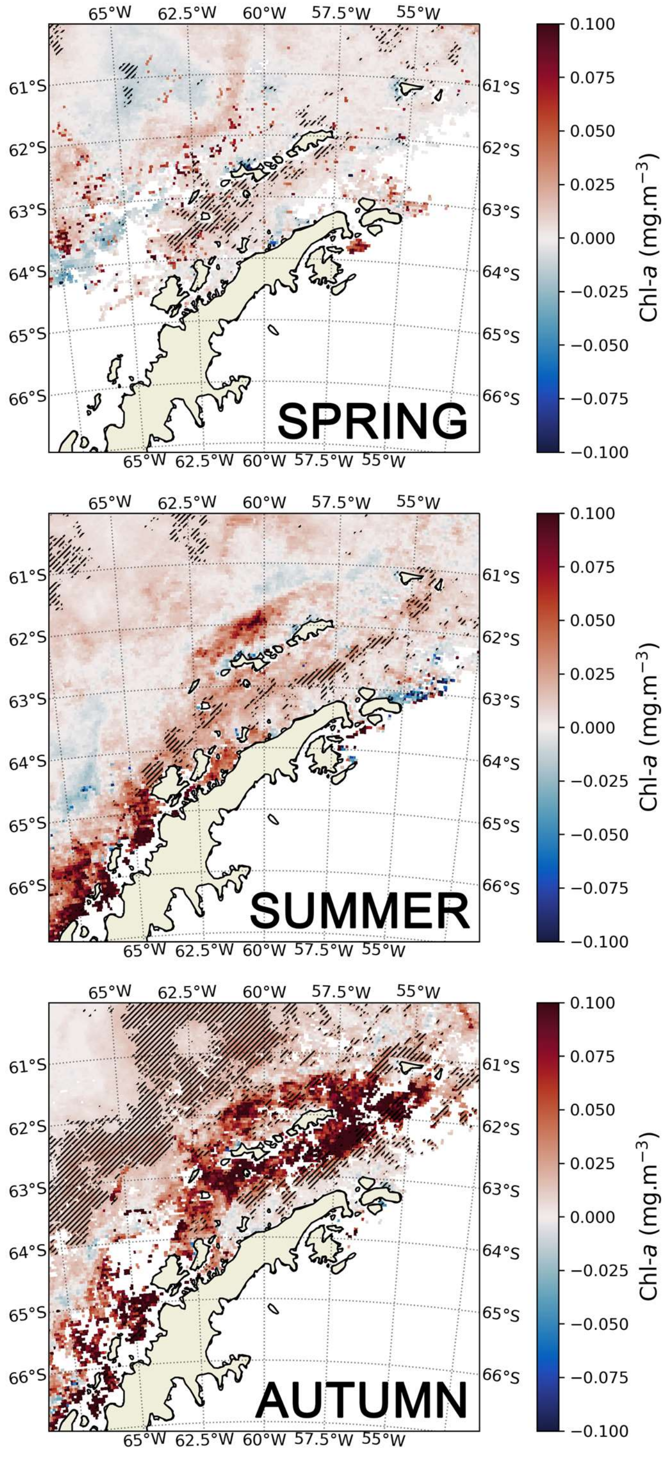

Sup. Fig. 3 | Satellite phytoplankton biomass increasing trends in the West Antarctic Peninsula. Calculated change in satellite-derived chlorophyll-*a* per year (mg m^{-3} $year^{-1}$) during spring (September-November), summer (December-February) and early spring (March-April) along the Antarctic Peninsula between 1998-2022. Oblique black lines represent the areas with statistically significant changes (p-value<0.05).

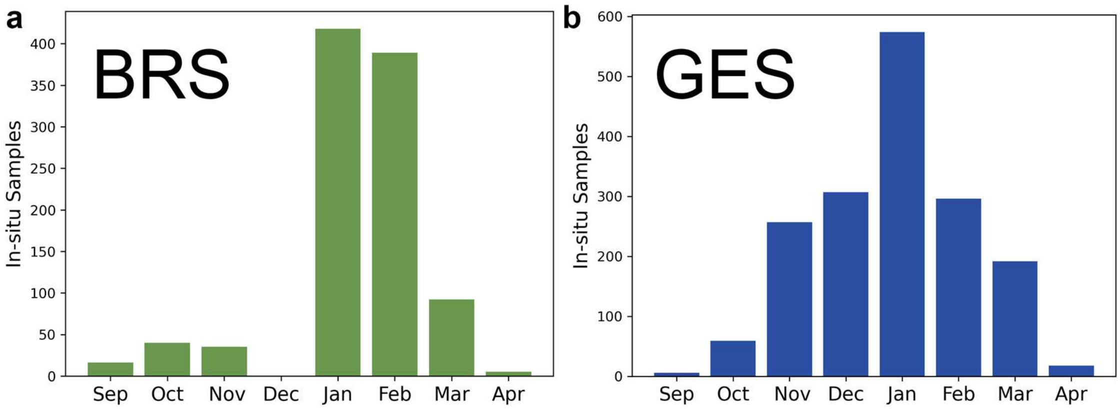

Sup. Fig. 4 | Seasonal distribution of in-situ samples showed in Figure 3b. Number of in-situ surface chlorophyll-a observations sampled each region (BRS: panel a; GES: panel b) and in each month between September and April 1998-2022.

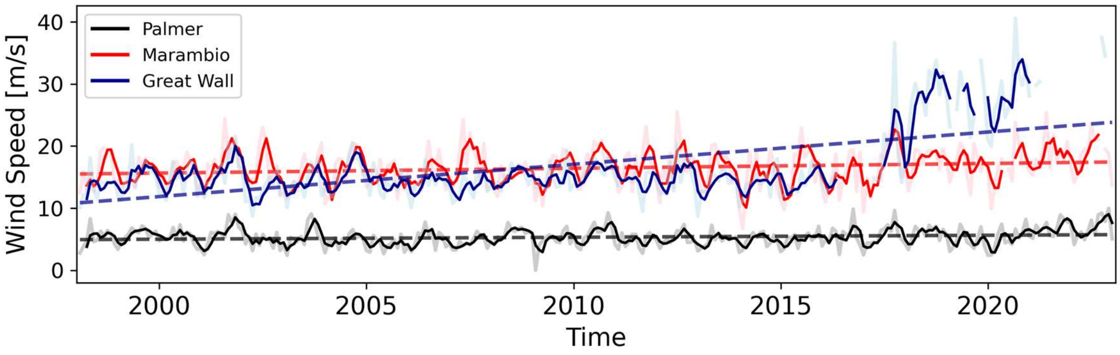

Sup. Fig. 5 | Linear trend in atmospheric wind speed during 1998-2022 in three stations in the Antartic Peninsula (Palmer, Marambio and Great Wall). Full lines represent the 6-month moving averages of the monthly-averaged wind speed measured at Palmer (64°46′27″S 64°03′10″W; black), Marambio (64°14′28″S 56°37′36″W; red), and Great Wall (62°13′01″S 58°57′43″W; blue) Stations. For each station, the shaded lines in background are the raw monthly-averaged wind speed. The dashed lines indicate the linear trend for each station, as follows: Palmer (y=0.003x + 4.93; R=0.15, p-value<0.01), Marambio (y=0.006x + 15.50; R=0.18, p-value<0.01), Great Wall (y=0.04x + 10.83, R=0.60, p-value<0.01).

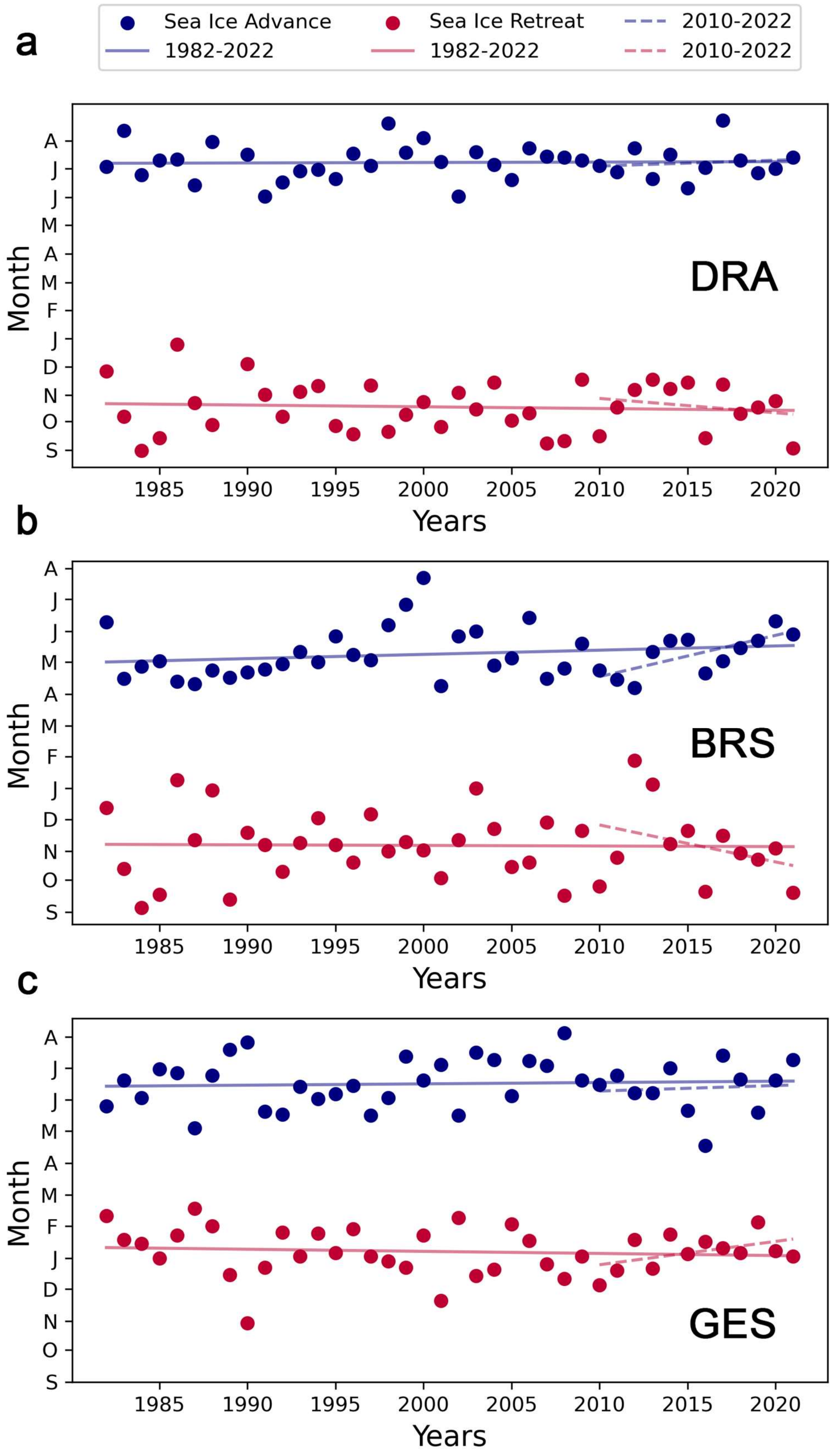

Sup. Fig. 6 | Trends in sea ice phenology across the Western Antarctic Peninsula. Interannual variability of Sea Ice Advance (blue) and Retreat (red) dates for the regions of the WAP - DRA (a), BRS (b) and GES (c) - during 1980 to 2022. Full lines correspond to the linear

regression for the 1982-2022 period, while the dashed lines correspond to the 2010-2022 period. These regions where chosen since they were the only ones that exhibited trends in biomass and/or bloom phenology (see Tables 1 and 2).

Sup. Table 1 | Statistical summary of summer chlorophyll-a and bloom phenology metrics during 1998-2022 for each region identified. CMean: mean December-February chlorophyll-a (Chl-*a*; mg m^{-3}) mean, C_{max}: Chl-a max, BPeak: Bloom Peak Date, Binit: Bloom Initiation Date, BTerm: Bloom Termination Date, BDur: Bloom Duration (weeks), BMag: Bloom Magnitude (mg m^{-3}; Simpson integrated Chl-a during the period of the bloom). For bloom phenology metrics, the standard deviation is always expressed in weeks. Note that BPeak, Binit and BTerm dates correspond to 8-day weeks, not individual days.

DRA	Chl-*a*	BPeak	BInit	BTerm	BDur	BMag
Mean	0.34	06/Dec	19/Oct	16/Feb	16	6.19
Min	0.05	19/Oct	09/Sep	14/Dec	8	2.09
Max	1.54	05/Apr	14/Dec	13/Apr	27	10.25
STDev	0.28	6	2	5	5	2
P10	0.11	-	-	-	9	3.10
P90	0.74	-	-	-	22	8.27
Mode	-	November	October	March	-	-
BRS	Chl-*a*	BPeak	BInit	BTerm	BDur	BMag
Mean	0.96	23/Jan	28/Nov	12/Mar	14	13.16
Min	0.06	20/Nov	04/Nov	15/Jan	9	6.19
Max	4.26	20/Mar	30/Dec	05/Apr	20	26.44
STDev	0.57	5	2	2	2	4.20
P10	0.40	-	-	-	12	9
P90	1.59	-	-	-	17	18.36
Mode	-	December	November	March	-	-
WED_N	Chl-*a*	BPeak	BInit	BTerm	BDur	BMag
Mean	0.78	14/Dec	12/Nov	16/Feb	13	9.94
Min	0.01	03/Oct	09/Sep	14/Dec	5	3.09
Max	5.58	04/Mar	30/Dec	28/Mar	24	20.25
STDev	0.68	4	4	3	4	4.35
P10	0.28	-	-	-	9	4.80
P90	1.55	-	-	-	16	14.68
Mode	-	December	November	January/March	-	-

Sup. Table 1 (Cont.) | Statistical summary of summer chlorophyll-a and bloom phenology metrics during 1998-2022 for each region identified. CMean: mean December-February chlorophyll-a (Chl-*a*; mg m^{-3}) mean, C_{max}: Chl-a max, BPeak: Bloom Peak Date, Binit: Bloom Initiation Date, BTerm: Bloom Termination Date, BDur: Bloom Duration (weeks), BMag: Bloom Magnitude (mg m^{-3}; Simpson integrated Chl-a during the period of the bloom). For bloom phenology metrics, the standard deviation is expressed in 8-day weeks. Note that BPeak, Binit and BTerm dates correspond to 8-day weeks, not individual days.

GES	Chl-*a*	BPeak	BInit	BTerm	BDur	BMag
Mean	2.11	31/Jan	06/Dec	12/Mar	13	28.30
Min	0.08	22/Dec	28/Nov	08/Feb	9	9.78
Max	12.14	20/Mar	22/Dec	28/Mar	15	77.08
STDev	1.94	3	1	1	2	17.24
P10	0.68	-	-	-	10	14.02
P90	4.41	-	-	-	15	53.52
Mode	-	January	December	March	-	-
WED_S	Chl-*a*	BPeak	BInit	BTerm	BDur	BMag
Mean	7.81	23/Jan	06/Dec	04/Mar	12	94.54
Min	0.09	12/Nov	17/Sep	31/Jan	6	9.30
Max	35.68	28/Mar	31/Jan	28/Mar	20	183.23
STDev	8.30	4	3	2	3	54.70
P10	0.57	-	-	-	10	21.18
P90	21.27	-	-	-	15	168.02
Mode	-	December	December	March	-	-

Sup. Table 2 | Statistical summary of the summer environmental conditions during 1998-2022 for each region identified. SST: sea surface temperature (°C), Sea Ice: sea ice concentration (%), PAR: photosynthetically active radiation (E m^{-2} d^{-1} $year^{-1}$), Wind Speed (m s^{-1}). Summer corresponds to December-February.

DRA	SST (ºC)	Sea Ice (%)	PAR (E m^{-2} d^{-1} $year^{-1}$)	Wind speed (m s^{-1})
Mean	1.73	0.13	34.38	6.23
Min	-0.65	0	7.68	0.04
Max	4.49	7.31	64.92	15.33
STDev	1.03	0.72	10.56	2.89
P10	0.33	0	20.98	2.74
P90	3.03	0.07	48.88	10.39
BRS	SST (ºC)	Sea Ice (%)	PAR (E m^{-2} d^{-1} $year^{-1}$)	Wind speed (m s^{-1})
Mean	0.84	1.20	33.19	6.01
Min	-0.97	0.01	6.15	0.21
Max	2.34	14.20	63.12	15.06
STDev	0.68	2.29	10.19	2.73
P10	-0.18	0.05	19.98	2.55
P90	1.62	3.67	47.05	9.70
WED_N	SST (ºC)	Sea Ice (%)	PAR (E m^{-2} d^{-1} $year^{-1}$)	Wind speed (m s^{-1})
Mean	-0.63	11.97	33.38	6.39
Min	-1.67	0	5.40	0.06
Max	0.53	81.50	64.71	16.06
STDev	0.49	17.38	12.55	3.07
P10	-1.35	0.28	17.80	2.44
P90	-0.01	40.83	51.85	10.61

Sup. Table 2 (Cont) | Statistical summary of the summer environmental conditions during 1998-2022 for each region identified. SST: sea surface temperature (°C), Sea Ice: sea ice concentration (%), PAR: photosynthetically active radiation (E m^{-2} d^{-1} $year^{-1}$), Wind Speed (m s^{-1}). Summer corresponds to December-February.

GES	SST (ºC)	Sea Ice (%)	PAR (E m^{-2} d^{-1} $year^{-1}$)	Wind speed (m s^{-1})
Mean	0.42	10.98	29.80	4.66
Min	-1.59	0.06	2.10	0.05
Max	2.82	81.02	64.66	14.57
STDev	0.95	15.49	14.21	2.50
P10	-1.00	0.96	12.70	1.57
P90	1.54	33.44	51.60	8.11
WED_S	SST (ºC)	Sea Ice (%)	PAR (E m^{-2} d^{-1} $year^{-1}$)	Wind speed (m s^{-1})
Mean	-0.76	27.84	16.39	2.79
Min	-1.80	0.02	1.60	0.04
Max	1.57	80.82	55.53	9.78
STDev	0.64	22.25	9.87	1.62
P10	-1.58	1.54	5.92	0.91
P90	0.02	61.77	29.89	5.02

Sup. Table 3 | Summary of the biological and environmental variables used in this work. Full names and detailed information on each product can be found in the Methods section. SST: sea surface temperature (°C), Sea Ice: sea ice concentration (%), PAR: photosynthetically active radiation (E m^{-2} d^{-1} $year^{-1}$), Wind Speed (m s^{-1}).

Variable	Units	Product/Source	Temporal Coverage	Spatial Resolution	Reference
Chlorophyll-*a* (satellite)	mg m^{-3}	ESA-CCI 6.0	1998-2022	4 km	1, 2
Chlorophyll-*a* (in-situ)	mg m^{-3}	GOAL, PALMER-LTER, Valente et al.	1998-2020	-	3, 4, 5
Sea Surface Temperature	ºC	OSTIA-L4	1998-2022	5 km	6
Sea Ice Concentration	%	OSTIA-L4	1982-2022	5 km	6
PAR	E m^{-2} d^{-1} $year^{-}$	NASA Globcolour	1998-2022	~8 km	7, 8
Wind Speed	m s^{-1}	ERA5 Reanalysis	1998-2022	~25 km	9
SAM	unitless	NOAA NCEP CPC	1998-2022	~8 km	10

*The meridional and zonal components of wind were acquired to calculate Wind Speed.
[1]Sathyendranath et al. 2019
[2]Ocean Colour Climate Change Initiative dataset, Version 6.0, European Space Agency, available online at http://www.esa-oceancolour-cci.org/.
[3]Palmer Station Antarctica LTER et al. 2022.
[4]Palmer Station Antarctica LTER et al. 2022.
[5]Valente et al. 2022.
[6]Good et al. 2020.
[7]Maritorena et al. 2010.
[8]Frouin et al. 2003.

[9]Hersbach et al. 2020.
[10]NOAA Monthly mean AO Index since January 1950, available at https://www.cpc.ncep.noaa.gov/.

Sup. Table 4 | P-values associated with the analysis of the yearly change in satellite-derived phytoplankton biomass within each month along the Antarctic Peninsula during 1998-2022 (Table 1). For each region, the p-value associated with the yearly linear change (mg m^{-3} year^{-}

[1]; slope of linear regression) in the spatially integrated mean chlorophyll-*a* is presented for each month between September and April, as well as for spring (September-November), summer (December-February), autumn (March-April) and full season (September-April). Bold *, **, and *** correspond to p-value<0.1, p-value<0.05, and p-value<0.01, respectively (a Pearson correlation test with a two-sided alternative hypothesis was used).

Period evaluated	DRA	BRS	WED_N	GES	WED_S
September	**0.010****	**0.001*****	0.649	0.211	**0.085***
October	**0.077***	**0.061***	0.201	0.140	0.312
November	0.929	0.300	0.761	0.170	0.971
December	0.416	0.407	0.257	**0.094***	0.147
January	0.152	0.427	0.416	0.274	0.565
February	0.123	0.145	0.477	0.395	0.231
March	**0.040****	**0.006*****	**0.032****	**0.005*****	**0.085***
April	**0.007*****	**0.001*****	**0.085***	-	-
Spring	0.503	**0.020****	0.268	0.150	0.682
Summer	0.140	0.224	0.427	0.192	0.101
Autumn	**0.013****	**0.002*****	**0.017****	**0.006*****	**0.068***
September-April	**0.068***	**0.019****	0.156	0.104	0.111

Sup. Table 5 | P-values associated with the analysis of the tearly change in phytoplankton bloom phenology along the Antarctic Peninsula during 1998-2022 (Table 2). For each region, the p-value associated with the yearly linear change (i.e., the slope of linear regression) was estimated for each bloom phenology metric calculated in this work: bloom initiation (weeks $year^{-1}$), bloom termination (weeks $year^{-1}$), bloom peak (weeks

year^{-1}), bloom duration (weeks year^{-1}), and bloom magnitude (mg m^{-3}year^{-1}; a measure of the biomass accumulated during the bloom). Bold *, **, and *** correspond to p-value<0.1, p-value<0.05, and p-value<0.01, respectively (a Pearson correlation test with a two-sided alternative hypothesis was used).

Phenology metric	DRA	BRS	GES
Bloom initiation	0.960	0.254	0.799
Bloom termination	**0.001*** **	0.471	**0.017** **
Bloom peak	0.573	0.394	0.832
Bloom duration	**0.002*** **	0.866	**0.058* **

References

1. Frouin, R. *et al.* The SeaWiFS PAR product. In *Algorithm Updates for the Fourth SeaWiFS Data Reprocessing*, S. B. Hooker and E. R. Firestone, Editors, CC NASA/TM-2003-206892, Vol. **22**, 46-50 (2003).

2. Good, S. *et al.* The current configuration of the OSTIA system for operational production of foundation sea surface temperature and ice concentration analyses. *Remote Sensing*, **12**(4), p.720 (2020).
3. Hersbach, H. *et al.* The ERA5 global reanalysis. *Quarterly Journal of the Royal Meteorological Society*, **146**(730), pp.1999-2049 (2020).
4. Maritorena, S. *et al.* Merged satellite ocean color data products using a bio-optical model: Characteristics, benefits and issues. *Remote Sensing of Environment*, **114**, 1791-1804 (2010).
5. Palmer Station Antarctica LTER *et al.* Photosynthetic pigments of water column samples and analyzed with High Performance Liquid Chromatography (HPLC), collected aboard Palmer LTER annual cruises off the coast of the Western Antarctica Peninsula, 1991 - 2020. ver 9. Environmental Data Initiative. https://doi.org/10.6073/pasta/9d0c1561ca8c5540227df6efa37c61b5 (2022).
6. Palmer Station Antarctica LTER *et al*. Photosynthetic pigments of water column samples analyzed using High Performance Liquid Chromatography (HPLC), sampled during the Palmer LTER field seasons at Palmer Station, Antarctica, 1991 – 2019. ver 7. Environmental Data Initiative. https://doi.org/10.6073/pasta/ffeb2f59fe11d8a0dabbb992e6b614e2 (2022).
7. Palmer Station Antarctica LTER and S. Stammerjohn. 2023. Sea ice duration or the time elapse between day of advance and day of retreat within a given sea ice year for the PAL LTER region West of the Antarctic Peninsula derived from passive microwave satellite, 1979 - 2023. ver 8. Environmental Data Initiative (2023).

8. Sathyendranath, S. *et al.* An Ocean-Colour Time Series for Use in Climate Studies: The Experience of the Ocean-Colour Climate Change Initiative (OC-CCI). *Sensors* **19**, 4285 (2019).
9. Valente, A. *et al.* A compilation of global bio-optical in situ data for ocean-colour satellite applications–version three. *Earth System Science Data* **14**, 5737-5770 (2022).
