# Supplementary material for: Climate change is associated with higher phytoplankton biomass and longer blooms in the West Antarctic Peninsula
Source: Nat Commun. 2024 Aug 2;15:6536. doi: 10.1038/s41467-024-50381-2 (PMC11297178; doi:10.1038/s41467-024-50381-2)
Supplement: Supplementary file 3 — Reporting Summary [file 41467_2024_50381_MOESM3_ESM.pdf]

Corresponding author(s): Afonso Ferreira

Last updated by author(s): Jun 28, 2024

Reporting Summary

Nature Portfolio wishes to improve the reproducibility of the work that we publish. This form provides structure for consistency and transparency in reporting. For further information on Nature Portfolio policies, see our Editorial Policies and the Editorial Policy Checklist.

Statistics

For all statistical analyses, confirm that the following items are present in the figure legend, table legend, main text, or Methods section.

n/a	Confirmed	
☒	☐	The exact sample size (*n*) for each experimental group/condition, given as a discrete number and unit of measurement
☒	☐	A statement on whether measurements were taken from distinct samples or whether the same sample was measured repeatedly
☒	☐	The statistical test(s) used AND whether they are one- or two-sided<br>*Only common tests should be described solely by name; describe more complex techniques in the Methods section.*
☒	☐	A description of all covariates tested
☒	☐	A description of any assumptions or corrections, such as tests of normality and adjustment for multiple comparisons
☒	☐	A full description of the statistical parameters including central tendency (e.g. means) or other basic estimates (e.g. regression coefficient) AND variation (e.g. standard deviation) or associated estimates of uncertainty (e.g. confidence intervals)
☒	☐	For null hypothesis testing, the test statistic (e.g. *F*, *t*, *r*) with confidence intervals, effect sizes, degrees of freedom and *P* value noted<br>*Give P values as exact values whenever suitable.*
☒	☐	For Bayesian analysis, information on the choice of priors and Markov chain Monte Carlo settings
☒	☐	For hierarchical and complex designs, identification of the appropriate level for tests and full reporting of outcomes
☒	☐	Estimates of effect sizes (e.g. Cohen's *d*, Pearson's *r*), indicating how they were calculated

Our web collection on statistics for biologists contains articles on many of the points above.

Software and code

Policy information about availability of computer code

Data collection	No code was used to collect data, only to process and analyse it.
Data analysis	All data processing and analysis were performed in Python 3.8.8. All custom code developed is now made publicly available in GitHub.

For manuscripts utilizing custom algorithms or software that are central to the research but not yet described in published literature, software must be made available to editors and reviewers. We strongly encourage code deposition in a community repository (e.g. GitHub). See the Nature Portfolio guidelines for submitting code & software for further information.

Data

Policy information about availability of data

All manuscripts must include a data availability statement. This statement should provide the following information, where applicable:

- Accession codes, unique identifiers, or web links for publicly available datasets
- A description of any restrictions on data availability
- For clinical datasets or third party data, please ensure that the statement adheres to our policy

The OC-CCI satellite chlorophyll-a product is available at https://www.oceancolour.org/. The OSTIA product is available at https://doi.org/10.48670/moi-00168. The GlobColour data is available at https://hermes.acri.fr/. The Southern Annular Mode (SAM) data is available at https://www.cpc.ncep.noaa.gov/). The in-situ chlorophyll-a data acquired from Valente et al. (2022) are available at https://doi.org/10.5194/essd-14-5737-2022. The in-situ chlorophyll-a data acquired from Palmer-LTER are available https://doi.org/10.6073/pasta/9d0c1561ca8c5540227df6efa37c61b5. The in-situ chlorophyll-a dataset from GOAL are available at https://zenodo.org/doi/10.5281/zenodo.12580624. The in-situ wind data from Palmer Station are available at https://doi.org/10.6073/

pasta/3eefb45dbfb784c3cabe3690ea46fe9e. The in-situ wind datasets from Marambio Station and Great Wall Station are available at https://www.ncei.noaa.gov/products/land-based-station/global-historical-climatology-network-daily. Source data are provided with this paper. All custom code developed within this work is available at: https://github.com/afonsomferreira/antarcticpeninsula-trends.

Research involving human participants, their data, or biological material

Policy information about studies with human participants or human data. See also policy information about sex, gender (identity/presentation), and sexual orientation and race, ethnicity and racism.

Reporting on sex and gender	NA
Reporting on race, ethnicity, or other socially relevant groupings	NA
Population characteristics	NA
Recruitment	NA
Ethics oversight	NA

Note that full information on the approval of the study protocol must also be provided in the manuscript.

Field-specific reporting

Please select the one below that is the best fit for your research. If you are not sure, read the appropriate sections before making your selection.

☐ Life sciences ☐ Behavioural & social sciences ☒ Ecological, evolutionary & environmental sciences

For a reference copy of the document with all sections, see nature.com/documents/nr-reporting-summary-flat.pdf

Ecological, evolutionary & environmental sciences study design

All studies must disclose on these points even when the disclosure is negative.

Study description	This study used environmental and ocean color satellite data and in-situ phytoplankton data to assess trends in coastal Antarctic phytoplankton. Satellite data was first analysed using a hierarchical clustering and then each cluster was the focus of more in-depth analysis. All of these were done in Python 3.8.8. In-situ chlorophyll-a data (as a proxy of phytoplankton biomass) was used, part of it was acquired from online public repositories (Palmer LTER), while the other part was kindly provided by GOAL-FURG. No experiments or treatments were performed at any stage of this study.
Research sample	Phytoplankton data was used, although all samples collected were natural samples (i.e. with mixed community) from the Antarctic Peninsula. No manipulation of the organisms were performed.
Sampling strategy	This study only used phytoplankton data measured at surface (5 m depth, at maximum).
Data collection	Data was collected using research vessels, mainly during the austral summer (December-February). Again, most of data used in this work is either already available publicly online (Palmer LTER) or has already been used in other studies by GOAL-FURG. The GOAL-FURG in-situ Chl-a measurements were collected during 12 austral summer expeditions to the WAP aboard the Brazilian vessels NP Almirante Maximiano and NP Ary Rongel (N = 533). Seawater samples (ranging from 0.5 to 2.5L) collected using Niskin bottles attached to a CTD Rosette were then filtered under low vacuum using GF/F filters (25 mm diameter, 0.7 µm pore size) and subsequently stored at -80ºC.
Timing and spatial scale	The in-situ samples analysed in this work were all collected along the marine ecosystems of the Antarctic Peninsula between 1997-2020. Nevertheless, the frequency and periodicity was not equal every year due to the complex logistics behind sampling in such a remote region as Antarctica.
Data exclusions	No data was excluded from the analysis.
Reproducibility	NA. No experiments or treatments were performed within this study.
Randomization	NA. No experiments or treatments were performed within this study.
Blinding	NA. No experiments or treatments were performed within this study.

Did the study involve field work? ☒ Yes ☐ No

Field work, collection and transport

Field conditions	The in-situ samples analysed in this work were all collected along the marine ecosystems of the Antarctic Peninsula between 1997-2020. As such, the environmental parameters were highly variable, but typically included near freezing temperatures.
Location	All in-situ samples were collected off the Western Antarctic Peninsula (West Antarctica), which exhibits a large variety of coastal marine ecosystems and spans over thousands of kilometers. Latitude and Longitude are provided, both in text and in Figures. All in-situ samples were collected at surface. This is also mentioned in the text.
Access & import/export	All sampling and personnel directly involved in it followed the guidelines of the Antarctica Treaty and operated under the Brazillian Polar Program.
Disturbance	The study cause no or negligible disturbance to the ecosystem.

Reporting for specific materials, systems and methods

We require information from authors about some types of materials, experimental systems and methods used in many studies. Here, indicate whether each material, system or method listed is relevant to your study. If you are not sure if a list item applies to your research, read the appropriate section before selecting a response.

Materials & experimental systems

n/a	Involved in the study
☒	☐ Antibodies
☒	☐ Eukaryotic cell lines
☒	☐ Palaeontology and archaeology
☒	☐ Animals and other organisms
☒	☐ Clinical data
☒	☐ Dual use research of concern
☒	☐ Plants

Methods

n/a	Involved in the study
☒	☐ ChIP-seq
☒	☐ Flow cytometry
☒	☐ MRI-based neuroimaging

Plants

Seed stocks	NA
Novel plant genotypes	NA
Authentication	NA
